# Pharyngeal High‐Resolution Manometry‐Based Evaluation of Dysphagia Recovery After Lateral Medullary Syndrome: A Case Series of Two Patients

**DOI:** 10.1002/ccr3.72201

**Published:** 2026-03-06

**Authors:** Hina Yoshida, Seiko Shibata, Yoko Inamoto, Ryusei Fukushima, Yoshitaka Wada, Yohei Otaka

**Affiliations:** ^1^ Department of Rehabilitation Medicine, School of Medicine Fujita Health University Toyoake Aichi Japan; ^2^ Faculty of Rehabilitation, School of Health Sciences Fujita Health University Toyoake Aichi Japan

**Keywords:** deglutition disorders, dysphagia, highresolution manometry, impedance, lateral medullary syndrome

## Abstract

To evaluate dysphagia, pharyngeal high‐resolution impedance manometry (P‐HRM‐I) is used in conjunction with videofluoroscopic examination of swallowing (VF) or videoendoscopic evaluation of swallowing to obtain additional objective data that cannot be captured by conventional assessment methods. Based on the Leuven Consensus of the International Pharyngeal Manometry Working Group for diagnosing pharyngeal and upper esophageal sphincter (UES) motility disorders, we present a case series of two patients illustrating the recovery process of dysphagia following lateral medullary syndrome. Two patients with severe dysphagia due to lateral medullary infarction caused by vertebral artery dissection were evaluated. In both patients, the initial P‐HRM‐I showed profound impairment of the UES opening and bolus passage, preventing oral intake. Balloon dilation of the UES, laryngeal elevation exercises, tongue strengthening exercises, and direct swallowing training were performed, and the functions of the pharynx and UES were regularly evaluated using VF and P‐HRM‐I. Following UES dilation, PHRM‐I revealed improved UES opening, enhanced pharyngeal contraction, and restoration of pharyngeal peristalsis. Both patients regained sufficient swallowing function to resume a regular diet. P‐HRM‐I may be a useful tool for quantitatively assessing UES function and bolus propulsion, identifying the pathophysiological components of dysphagia, guiding individualized treatment, and monitoring post‐intervention recovery.

## Key Clinical Message

Serial P‐HRM‐I assessments quantified UES dysfunction and pharyngeal motility recovery after lateral medullary syndrome. Guided by the Leuven Consensus, P‐HRM‐I provided standardized interpretation and, complementing VFSS, sensitively monitored therapeutic response and swallowing restoration in neurogenic dysphagia.

## 1 Introduction

Videofluoroscopic examinations of swallowing (VF) and videoendoscopic evaluation of swallowing (VE) are widely used to assess dysphagia. However, these imaging‐based modalities do not allow for quantitative analysis of the biomechanical aspects of swallowing.

Pharyngeal high‐resolution impedance manometry (P‐HRM‐I), which combines high‐resolution manometry (HRM) with impedance, enables objective assessment of pharyngeal and upper esophageal sphincter (UES) function, including analysis of bolus propulsion and UES relaxation and opening [1, 2, 3, 4, 5].

In 2025, the International Pharyngeal Manometry Working Group introduced a standardized diagnostic framework for the evaluation of pharyngeal and UES motility disorders using P‐HRM‐I [6]. This framework known as the Leuven Consensus. According to this consensus, UES relaxation is assessed using UES integrated relaxation pressure (IRP) and UES relaxation time (RT), whereas UES opening is evaluated using UES maximum admittance (MaxAd) and hypopharyngeal intrabolus pressure (IBP). Together, these metrics allow separate evaluation of UES relaxation and opening mechanics. Pharyngeal contractile strength is measured as the integrated pressure across the nasopharynx, oropharynx, and hypopharynx, along with the hypopharyngeal peak pressure (HPeakP), which reflects the bolus‐driving force. Moreover, impaired UES opening is associated with characteristic pharyngeal pressure topography patterns that have been systematically categorized.

Dysphagia associated with lateral medullary syndrome (LMS) typically involves severe impairment of the pharyngeal phase during swallowing. A hallmark feature is UES dysfunction, particularly cricopharyngeal muscle relaxation failure on the affected side, often accompanied by pharyngeal muscle paralysis [7, 8, 9]. UES balloon dilation is an effective therapeutic approach in such cases [10]. In this study, we aimed to elucidate the course of swallowing function recovery in two patients with LMS‐related dysphagia who underwent UES balloon dilation. The VF and P‐HRM‐I data were analyzed using the Leuven Consensus. There are limited studies that objectively quantify swallowing biomechanics in LMS‐related dysphagia using the standardized Leuven Consensus framework. In particular, longitudinal data describing swallowing function recovery after UES balloon dilation, analyzed with P‐HRM‐I, are scarce. This limits our understanding of treatment response and the mechanisms underlying recovery in these patients. Therefore, this study applies Leuven Consensus–based P‐HRM‐I analysis, in combination with VF, to comprehensively evaluate swallowing function changes in LMS‐related dysphagia before and after UES balloon dilation.

## 2 Case Presentation

### 2.1 Case 1

A 52‐year‐old female presented with headache, dizziness, dysphagia, gait disturbance, and decreased pain and temperature sensation on the right side of her face and the left side of her body. Magnetic resonance imaging (MRI) revealed a right lateral medullary infarction due to vertebral artery dissection, and the patient was admitted to another hospital. A 3 mL water swallow test raised the suspicion of aspiration, and on day 11 after onset, swallowing function was evaluated. VF revealed that the bolus failed to pass through the UES, with a significant residue in the pyriform sinus, indicating UES dysfunction. Consequently, sustained UES dilation training using a double‐balloon catheter (Create Medic, Yokohama, Japan) was initiated (dilation volume, 20 mL; dilation duration, 1 min × 3 sessions).

Upon transfer to our hospital on day 24 post‐onset, VE revealed weak movement of the right soft palate, right vocal fold paralysis, and saliva pooling in the right pyriform sinus. UES balloon dilation training was continued, along with laryngeal elevation exercises, including tongue base elevation, Shaker exercises, and tongue dexterity training, to enhance bolus propulsion.

P‐HRM‐I was performed concurrently with VF using a 36‐channel solid‐state HRM‐impedance catheter (outer diameter 4.2 mm; Unisensor AG, Attikon, Switzerland). HRM data were analyzed using STARLET software (STARMEDICAL Inc., Tokyo, Japan) on days 27, 48, and 83 post‐onset.

Individual swallows were identified and manually selected from pressure topography plots synchronized with VF images. For each swallow, predefined anatomical landmarks were manually assigned, including the velopharynx, mesopharynx, hypopharynx, and UES. Following landmark assignment, swallow‐specific pressure metrics were calculated automatically by the software.

The landmark definitions and analysis workflow were based on the standardized Swallow Gateway framework for pharyngeal HRM analysis, ensuring consistency with previously published P‐HRM‐I studies (Table S1).

**FIGURE 1:**
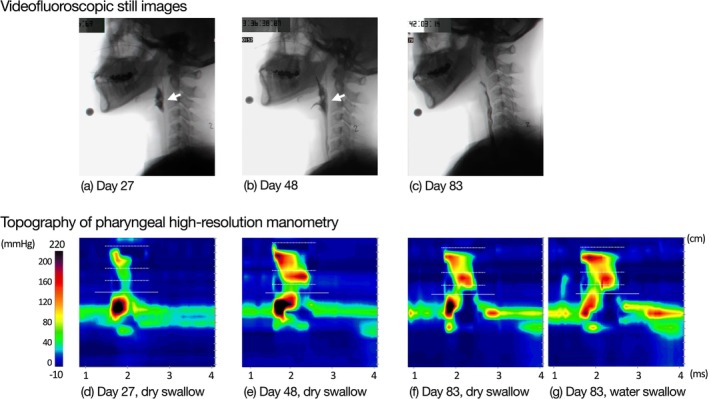
Swallowing evaluations in Case 1. Upper panels show videofluoroscopic still images obtained at the point of maximal laryngeal elevation during swallowing. (a) On day 27, the bolus did not pass through the upper esophageal sphincter (UES), and pharyngeal residue was observed (white arrow). (b) On day 48, partial bolus passage through the UES was observed, with residual material remaining in the pharynx (white arrow). (c) On day 83, bolus passage through the UES was observed, and no pharyngeal residue was present. Lower panels show representative pressure topographs of dry swallows on each evaluation day (d–f) and a 4‐mL viscous water swallow (International Dysphagia Diet Standardization Initiative [IDDSI] level 3) on day 83 (g). Pressure patterns differed across evaluation days, with pan‐pressurization on day 27 (d), distal compartmentalized pressurization on day 48 (e), and transient bolus pressurization on day 83 (f, g). Solid lines indicate the UES apogee; dotted lines indicate the margins of the velopharynx, mesopharynx, and hypopharynx.

The definitions of the P‐HRM‐I parameters were adopted from the Leuven framework, and normative values were obtained from previously published literature. The VFSS and P‐HRM‐I images (Figure 1) and P‐HRM‐I results (Table 1) are presented.

**TABLE 1:** High‐resolution manometry‐impedance results of Case 1.

	Parameters	Day 27	Day 48	Day 83	
		Dry swallow	Dry swallow	Dry swallow	Water swallow
UES metric	UES IRP, mmHg	46.1 (12.8)	27.8 (1.4)	−6.5 (1.1)	2.2 (9.5)
	UES RT, ms	21.3 (14.4)	158.7 (18.9)	346.0 (36.4)	494.0 (8.5)
	UES MaxAd, mS	0.8	1.7 (0.2)	2.9 (0.2)	4.0 (0.3)
	IBP, mmHg	—	—	—	82.2 (1.3)
Pharyngeal metric	VCI, mmHg.s. Cm	110.9 (5.6)	134.3 (12.4)	176.7 (21.3)	104.5 (14.8)
	MCI, mmHg.s. Cm	52.2 (4.0)	132.0 (10.0)	170.2 (1.4)	163.5 (13.4)
	HPCI, mmHg.s. Cm	—	1.0 (0.0)	6.0 (3.0)	1.5 (0.7)
	HPeak P, mmHg	—	—	42.7 (2.6)	32.4 (1.3)

*Note:* Values are presented as mean (standard deviation), where applicable. For parameters with only a single measurement, the individual values are shown without averaging.

Abbreviations: HPCI, hypopharyngeal contractile integral; HPeak P, hypopharyngeal peak pressure; IBP, hypopharyngeal intrabolus pressure; IRP, integrated relaxation pressure; MaxAd, maximum admittance; MCI, mesopharyngeal contractile integral; RT, relaxation time; UES, upper esophageal sphincter; VCI, velopharyngeal contractile integral.

On day 27, VF revealed failure of bolus passage through the UES, with a large amount of pharyngeal residue (Figure 1a). Because aspiration was suspected, P‐HRM‐I was limited to the assessment of spontaneous swallowing of saliva. UES metrics showed a markedly elevated mean (standard deviation, SD) UES IRP of 46.1 (12.8) mmHg, indicating impaired relaxation. The mean (SD) UES RT was short at 21.3 (14.4) ms, and the UES MaxAd was low at 0.8 mS, both indicating impaired UES opening. Pharyngeal metrics revealed that the mean (SD) velopharyngeal contractile integral (VCI) was near the normal range at 110.9 (5.6) mmHg·s·cm, but the mean (SD) mesopharyngeal contractile integral (MCI) was reduced to 52.2 (4.0) mmHg·s·cm, and the hypopharyngeal contractile integral (HPCI) could not be measured, reflecting weakened contraction of the middle and lower pharynx. Therefore, no pharyngeal peristaltic waves were generated, and the bolus pressure pattern corresponded to the pan‐pressurization type (type 1) (Figure 1d). Oral intake was deemed unsafe; therefore, continuous balloon dilatation therapy and other indirect training were continued.

On day 48, VF showed partial bolus passage through the UES (Figure 1, b); however, owing to the continued high aspiration risk, saliva swallowing was again assessed using P‐HRM‐I. The mean (SD) UES IRP decreased to 27.8 (1.4) mmHg, whereas the mean (SD) UES RT and UES MaxAd increased to 158.7 (18.9) mS and 1.7 (0.2) mS, respectively, showing partial improvement in the UES function. The mean (SD) MCI increased to 132.0 (10.0) mmHg·s·cm. The bolus pressure pattern changed to a distal compartmentalized pressurization pattern (type 2) (Figure 1e). Stepwise oral intake training using safe food textures was initiated.

On day 66, the patient consumed three meals of texture‐modified food per day, and UES balloon dilation therapy was completed. On day 83, VF showed good pharyngeal bolus passage without laryngeal penetration or pharyngeal residue (Figure 1c). P‐HRM‐I was performed with dry swallows and 4 mL of water swallows (International Dysphagia Diet Standardization Initiative [IDDSI] levels 3 [11]). For dry and water swallows, the mean (SD) UES IRPs were within normal ranges at −6.5 (1.1) mmHg and 2.2 (9.5) mmHg, respectively. The mean (SD) UES RTs were 346.0 (36.4) ms and 494.0 (8.5) ms, and the mean (SD) UES MaxAd values were 2.9 (0.2) mS and 4.0 (0.3) mS, respectively, with water swallowing reaching normal values. The IBP, which indicates resistance to UES passage, remained elevated. For pharyngeal metrics, the VCI and MCI exceeded the normal values, the HPCI was low. HPeakP, representing bolus‐driving pressure, remained below normal during water swallowing, and pharyngeal peristalsis was incomplete. The bolus pressure pattern shifted to transient pressurization (type 3) (Figure 1f,g). Following this evaluation, the patient began drinking water from a cup and was discharged on a regular diet 98 days post‐onset.

### 2.2 Case 2

**FIGURE 2:**
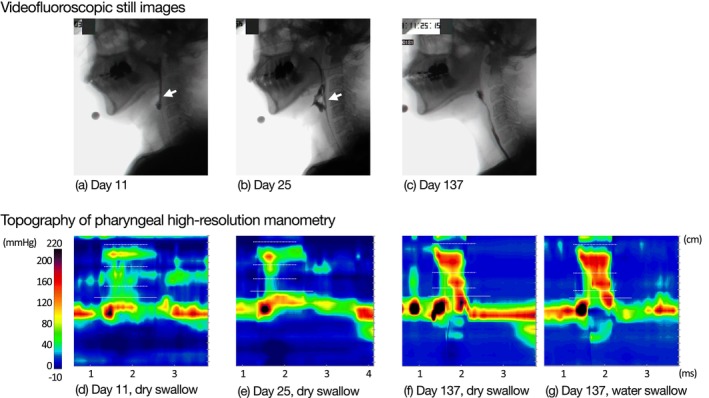
Swallowing evaluations in Case 2. Upper panels show videofluoroscopic still images obtained at the point of maximal laryngeal elevation during swallowing. (a) On day 11, the bolus did not pass through the upper esophageal sphincter (UES) and remained in the pharynx. (b) On day 25, limited bolus passage through the UES was observed; incomplete pharyngeal cavity collapse was noted (white arrow). (c) On day 137, pharyngeal contraction was observed, with bolus passage through the UES. Lower panels show representative pressure topographs of dry swallows on each evaluation day (d–f) and a 4‐mL viscous water swallow (IDDSI level 3) on day 137 (g). Pan‐pressurization patterns were observed on days 11 (d) and 25 (e), whereas distal compartmentalized pressurization was observed on day 137 (f, g), with pharyngeal stripping waves present. Solid lines indicate the UES apogee; dotted lines indicate the margins of the velopharynx, mesopharynx, and hypopharynx.

A 58‐year‐old male was admitted to our hospital with headache, dysarthria, and dysphagia. MRI revealed a right lateral medullary infarction due to vertebral artery dissection. Physical examination revealed decreased pain and temperature sensation on the right side of the face and left side of the body, right‐sided ptosis, rightward deviation of the tongue, nasal speech, breathy hoarseness, and ataxia of the right upper and lower limbs. A 3 mL water swallow test revealed coughing, raising the suspicion of aspiration. VE showed right‐sided vocal fold paralysis. VF and P‐HRM‐I assessments were conducted on days 11, 25, and 137 after stroke onset (Figure 2; Table 2).

**TABLE 2:** High‐resolution manometry‐impedance results of Case 2.

	Parameters	Day 11	Day 25	Day 137	
		Dry swallow	Dry swallow	Dry swallow	Water swallow
UES metric	UES IRP, mmHg	51.7 (16.9)	40.5 (10.9)	90.1 (13.8)	66.0 (45.6)
	UES RT, ms	—	79.7 (115.5)	—	84.8 (90.1)
	UES MaxAd, mS	—	0.6	—	4.5 (0.1)
	IBP, mmHg			—	51.7 (10.0)
Pharyngeal metric	VCI, mmHg.s. Cm	80.0 (16.0)	70.3 (17.5)	165.3 (38.0)	160.5 (9.2)
	MCI, mmHg.s. Cm	81.3 (43.5)	22.7 (11.6)	122.3 (21.0)	173.5 (12.0)
	HPCI, mmHg.s. Cm	—	—	101.3 (22.1)	69.5 (10.6)
	HPeak P, mmHg	—	—	207.4 (36.4)	143.7 (11.7)

*Note:* Values are presented as means (standard deviations), where applicable. For parameters with only a single measurement, individual values are shown without averaging.

Abbreviations: HPCI, hypopharyngeal contractile integral; HPeak P, hypopharyngeal peak pressure; IBP, hypopharyngeal intrabolus pressure; IRP, integrated relaxation pressure; MaxAd, maximum admittance; MCI, mesopharyngeal contractile integral; RT, relaxation time; UES, upper esophageal sphincter; VCI, velopharyngeal contractile integral.

On day 11, the VF showed that the bolus did not pass through the pharynx, and large amounts of residue remained in the pyriform sinus, accompanied by aspiration (Figure 2a). Swallowing in the right head rotation position reduced aspiration, but not completely. P‐HRM‐I was performed only with saliva swallowing. UES metrics showed a high mean (SD) UES IRP of 51.7 (16.9) mmHg, and the UES MaxAd and UES RT were unmeasurable, indicating impaired UES relaxation and opening. Pharyngeal metrics showed a mildly reduced mean (SD) VCI of 80.0 (16.0) mmHg·s·cm, a normal mean (SD) MCI of 81.3 (43.5) mmHg·s·cm, and an unmeasurable HPCI.

The bolus pressure waveform exhibited type 1, indicating the absence of middle‐to‐lower pharyngeal contraction required to generate pharyngeal peristalsis (Figure 2d). The following day, double‐balloon dilation training for UES dysfunction (expansion volume, 20 mL; duration, 1 min × 3 times) was initiated, along with tongue base elevation exercises and the Mendelsohn maneuver to improve laryngeal elevation and pharyngeal contraction.

On day 25 post‐onset, the mean (SD) UES IRP improved from 51.7 (16.9) mmHg to 40.5 (10.9) mmHg, the UES RT improved from unmeasurable to 70.7 (115.5) ms, and the UES MaxAd slightly improved from unmeasurable to 0.6 mS.

In contrast, pharyngeal metrics remained low, and the bolus pressure waveform was still of type 1 (Figure 2e). VF in the neutral head position showed restricted UES passage with laryngeal penetration and pharyngeal residue (Figure 2b); however, in the right head rotation position, UES passage was achieved without aspiration. Because bolus passage was possible in the rotated head position, oral intake of texture‐modified food was initiated under postural adjustment, and by day 40, the patient was able to consume three meals per day. On day 48, VE showed resolution of pharyngeal residue, and UES balloon dilation therapy was discontinued. By day 53, the patient was able to consume a regular diet within 30 min in the neutral head position, and he was discharged home on day 70.

On day 137 post‐onset, VF showed that the bolus passed through the UES in the neutral head position without aspiration or pharyngeal residue (Figure 2c). P‐HRM‐I was performed for both saliva swallowing and 4 mL water swallowing (IDDSI levels 3). The mean (SD) UES IRP increased from 51.7 (16.9) mmHg at baseline to 90.1 (13.8) mmHg for saliva swallowing, and was 66.0 (45.6) mmHg for water swallowing, indicating elevated values; however, the mean (SD) UES MaxAd was 4.5 (0.1) mS, confirming adequate UES opening. Pharyngeal metrics improved from the first P‐HRM‐I: the mean (SD) VCI increased from 80.0 (16.0) mmHg·s·cm to 160.5 (9.2), MCI from 81.3 (43.5) mmHg·s·cm to 175.3 (12.0), HPCI from unmeasurable to 69.5 (10.6) mmHg·s·cm, and HPeakP from unmeasured at baseline to 143.7 (11.7) mmHg, all within or above normal ranges.

The bolus pressure waveform changed from type 1 to type 2, suggesting improvement in UES opening and recovery of pharyngeal contractile function (Figure 2f,g).

## 3 Differential Diagnosis

In both patients, dysphagia due to structural esophageal stenosis, neurodegenerative disease, and peripheral neuromuscular disorders was considered. Imaging findings and neurological examination supported the diagnosis of lateral medullary syndrome caused by vertebral artery dissection as the primary etiology of dysphagia.

## 4 Conclusion and Results (Outcome and Follow‐Up)

Both patients showed gradual recovery of swallowing function following UES balloon dilation and structured swallowing rehabilitation. Serial P‐HRM‐I assessments demonstrated improvement in UES opening and pharyngeal contractility, enabling resumption of a regular diet in both cases.

## 5 Discussion

We examined the recovery process in two patients with dysphagia associated with LMS who underwent swallowing rehabilitation primarily involving balloon dilatation of UES, using VF and P‐HRM‐I. Serial P‐HRM‐I measurements allowed observation of temporal changes in UES function and pharyngeal contractility in parallel with changes in bolus passage. In addition, classification of bolus pressurization patterns according to the Leuven Consensus was applied to describe pharyngeal pressure patterns observed during the clinical course. This framework facilitated a structured description of pharyngeal pressure characteristics across different time points during rehabilitation.

### 5.1 Upper Esophageal Sphincter Balloon Dilation

**FIGURE 3:**
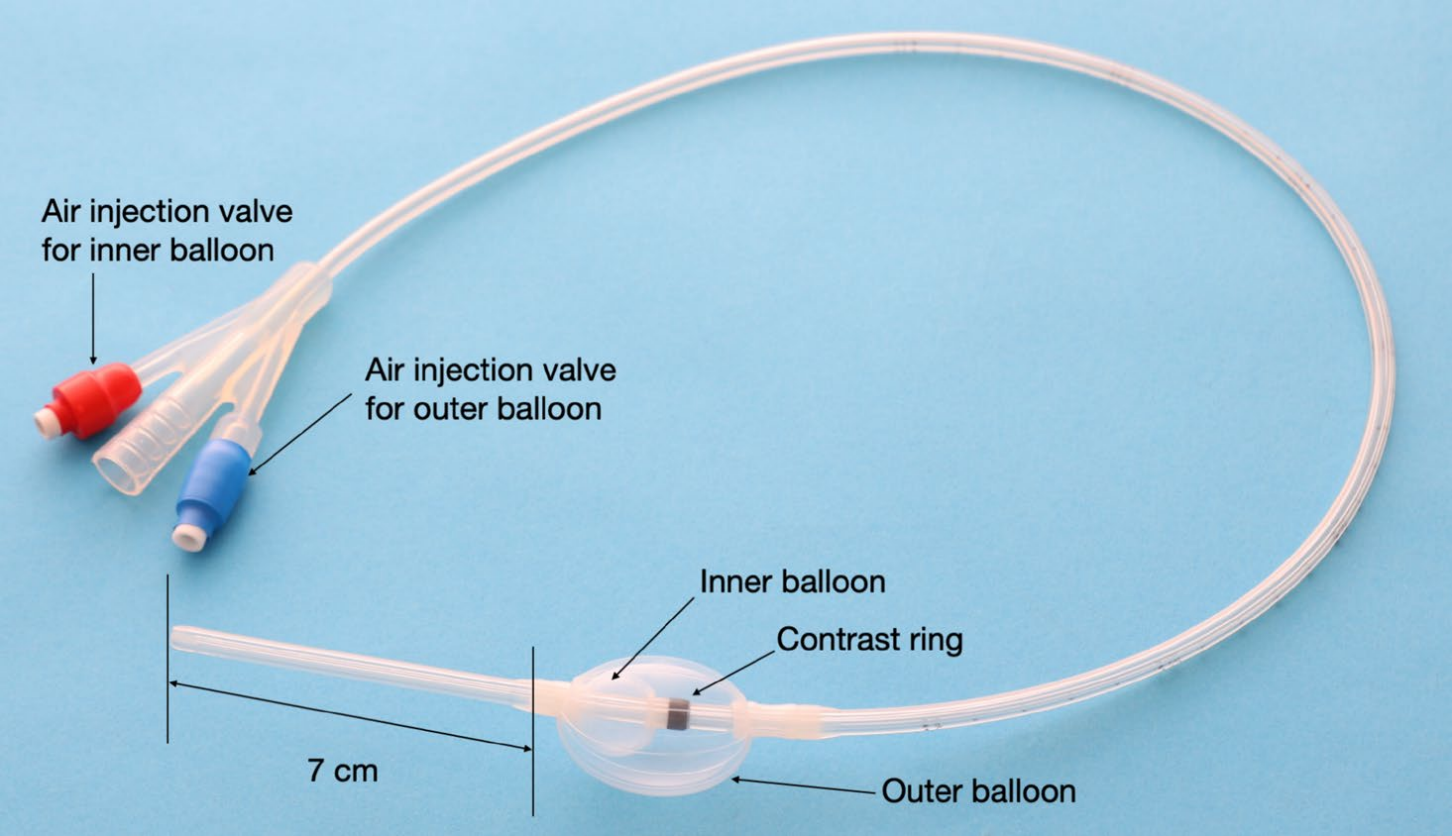
Double‐balloon catheter for esophageal inlet dilatation. The double‐balloon catheter used for esophageal inlet dilatation (Create Medic, Yokohama, Japan) consists of a 14‐Fr silicone catheter with two balloons positioned 7 cm from the tip. The inner spherical balloon is positioned distal to the upper esophageal sphincter (UES), and the outer oval balloon is used for UES dilatation. These captions were included in the manuscript; however, we are providing them here again for clarity.

The balloon catheter used in this study was an elliptical double‐balloon device (Figure 3) specifically designed to conform to the UES anatomy and allow for continuous dilation. UES balloon dilation therapy was initiated on day 11 post‐onset in Case 1 and day 12 in Case 2. The procedure was performed thrice daily, with each session consisting of 1 min of continuous dilation and continued until functional oral intake was achieved.

UES balloon dilation has been applied not only for UES dysfunction due to brainstem lesions but also for structural stenosis, such as esophageal webs or post‐radiation strictures [12]. Inadequate opening of the UES produces increases intrapharyngeal pressure during swallowing, and prolonged inadequate opening may cause pressure injury to the pharyngeal muscles [13]. In the present cases, early initiation and continuous balloon dilation therapy contributed to improvement in UES opening and prevention of pharyngeal muscle compression injury, which may have led to improvement in pharyngeal function.

### 5.2 UES Relaxation and Opening

In P‐HRM‐I, *UES IRP* and *UES RT* were used as relaxation indices of the UES, whereas *UES MaxAd* and *IBP* serve as indices of the UES opening [6]. *IBP* is the peak hypopharyngeal pressure measured during bolus transit and reflects the resistance the bolus encounters at the UES; therefore, IBP increases when the UES opening is insufficient. Because severe aspiration initially precluded bolus swallowing, IBP could only be examined during the final assessment with water swallowing.

In Case 1, during the first evaluation, all three UES indices were abnormal, indicating deficits in both UES relaxation and opening. UES function improved over time, and by day 83, the UES IRP had normalized. Although UES RT and UES MaxAd remained low during saliva swallowing, they were within normal limits during water swallowing. These findings suggest that bolus‐driving pressure plays a crucial role in facilitating UES opening.

Among the three contributing factors—UES relaxation, laryngeal elevation, and bolus‐driving pressure—bolus‐driving pressure may be particularly important.

In Case 2, even on day 137, when the patient was able to eat a regular diet, the UES IRP and IBP were still high, and the UES RT was short, indicating a persistent impairment of UES relaxation. Nevertheless, UES MaxAd during bolus swallowing was within the normal range. Given the concurrently elevated VCI and MCI values, we inferred that the increased pharyngeal intraluminal pressure compensated for impaired UES relaxation by augmenting the bolus‐driving pressure.

### 5.3 Bolus Pressurization Patterns

In cases of impaired UES relaxation or opening, bolus resistance at the level of the UES increases. On P‐HRM‐I topography, this is visualized as a pressurization pattern within the pharyngeal lumen during bolus transit. According to the Leuven Consensus, these bolus pressurization patterns are classified into three types based on topographic images [6]. Pan‐pressurization type (type 1) and distal compartmentalized pressurization type (type 2) are characterized by sustained resistance at the UES level during bolus passage through the pharynx. These types may reflect abnormalities in the timing of cricopharyngeal muscle relaxation, structural limitations in the UES opening, dysfunctions such as poor bolus propulsion by the tongue, and impaired laryngeal elevation. Type 1 represents a more severe condition with pronounced impairment of UES relaxation and opening, resulting in elevated pressure throughout the pharynx. In contrast, type 2 indicates less severe dysfunction, with pressure elevation localized primarily in the hypopharynx. The transient pressurization type (type 3) is thought to reflect transient hyperactivity of the cricopharyngeal muscle during swallowing or a delayed cessation of its activity. Consequently, pharyngeal contractions become desynchronized with UES relaxation, leading to a transient intrapharyngeal pressure elevation. In the two present cases, both initially demonstrated type 1 patterns due to significant UES relaxation impairment. With the improvement in UES relaxation and opening, the pressurization pattern changed to type 3 in Case 1 and type 2 in Case 2. However, none of the patients returned to a normal pattern, suggesting that the impairment of the UES opening persisted.

### 5.4 Pharyngeal Contraction

Bolus propulsion through the pharynx is primarily driven by the tongue, whereas the pharyngeal constrictor muscles clear the residual material. When the bolus volume is small, the coordinated pharyngeal peristaltic wave generated by the tongue and constrictor muscles is essential for bolus propulsion and clearance. In P‐HRM‐I topography, this peristaltic wave appears as a continuous pressure sequence across the nasopharynx, mesopharynx, and hypopharynx. In both cases, the MCI and HPCI contractile integrals were markedly low, indicating pharyngeal hypocontractility due to muscle paresis. Over time, mesopharyngeal contraction strengthened, and a pharyngeal peristaltic wave became evident, suggesting recovery of the pharyngeal constrictor muscles. When the peristaltic wave appeared, the lateral view of VF showed no laryngeal penetration, and both patients progressed to a regular diet. MCI in Case 1 and both VCI and MCI in Case 2 were markedly elevated compared with the normative values. This was presumed to be a compensatory response to the increased IBP observed in both cases, reflecting the resistance to bolus passage through the UES.

The Leuven Consensus states that bolus tasks should employ volumes as large as the patients can tolerate [6]. In these cases, severe early aspiration limited the evaluation of saliva swallows, and even after bolus intake became possible, testing was restricted to volumes that would not provoke aspiration; thus, the bolus volumes were insufficient to fully assess the entire swallowing apparatus.

In the present study, statistical analysis was not feasible because the sample size was limited to two patients. In addition, the range of disease severity in the study population was narrow, and there were minor differences in the timing of evaluations. Furthermore, P‐HRM‐I assessments were performed primarily during saliva swallows and under conditions involving limited bolus volumes, which may not fully reflect swallowing function across a broader range of bolus consistencies and volumes. Moreover, as this study represents a two‐case series, caution is required when interpreting the findings and extrapolating them to the broader population of patients with dysphagia associated with LMS.

Nevertheless, interpretation of P‐HRM‐I findings based on the Leuven Consensus provided a structured framework for describing components of dysphagia, and longitudinal assessment allowed detailed documentation of changes in swallowing function during the recovery process associated with LMS.

## Author Contributions

**Hina Yoshida:** investigation, visualization, writing – original draft. **Seiko Shibata:** data curation, formal analysis, visualization, writing – review and editing. **Yoko Inamoto:** data curation, formal analysis, writing – review and editing. **Ryusei Fukushima:** investigation. **Yoshitaka Wada:** investigation. **Yohei Otaka:** supervision.

## Funding

The authors have nothing to report.

## Ethics Statement

Ethical approval was waived according to the policy of our institution for case reports.

## Consent

Written informed consent was obtained from each patient for publication of this case series and accompanying images.

## Conflicts of Interest

The authors declare no conflicts of interest.

## Supporting information


**Table S1:** Swallow function metrics of high‐resolution manometry.

## Data Availability

The data that support the findings of this case report are available from the corresponding author upon reasonable request.
