# Supplementary material for: Pharyngeal High‐Resolution Manometry‐Based Evaluation of Dysphagia Recovery After Lateral Medullary Syndrome: A Case Series of Two Patients
Source: Clin Case Rep. 2026 Mar 6;14(3):e72201. doi: 10.1002/ccr3.72201 (PMC12964158; doi:10.1002/ccr3.72201)
Supplement: Supplementary file 1 — Table S1: Swallow function metrics of high‐resolution manometry. [file CCR3-14-e72201-s001.pdf]

Supplementary Table Swallow function metrics of high-resolution manometry

Metric	Abbreviation	Definition	Reference values (Abnormal criteria or normative mean [95% CI])
**UES metrics: UES relaxation and opening**			
UES integrated relaxation pressure	UES IRP, mmHg	UES IRP indicates UES relaxation, calculated as the median of the lowest 0.20–0.25 s e-sleeve pressure.	Abnormal criteria >2.9 [1]<br>–2.75 [–3.53 to 1.968] [2]<br>4.95 [3.69–6.21] [3]
UES relaxation time	UES RT, ms	UES RT indicates the duration of UES relaxation, defined as the e-sleeve pressure interval below 50% of baseline or 35 mmHg.	Abnormal criteria <410 [1]<br>749.09 [562.06–936.12] [2]
UES maximum admittance	UES MaxAd, mS	UES MaxAd reflects UES opening, defined as the peak admittance during trans-sphincteric bolus flow.	Abnormal criteria <3.6 [1]
Hypopharyngeal intrabolus pressure	IBP, mmHg	IBP is the pressure 1 cm above the UES apex at maximal hypopharyngeal distension from impedance.	Abnormal criteria >17.0 [1]
**Pharyngeal metrics: pharyngeal contractile strength**			
Velopharyngeal contractile integral	VCI, mmHg.s. cm	VCI quantifies contractile vigor in the velopharyngeal region as the mean pressure multiplied by its duration and length.	Abnormal criteria <18.4[1]<br>130.32 [118.2–142.4][3]
Mesopharyngeal contractile integral	MCI, mmHg.s. cm	MCI quantifies contractile vigor in the mesopharyngeal region as the mean pressure within a space–time box multiplied by its duration and length.	Abnormal criteria <41.0 [1]<br>76.06 [67.99–84.13)] [3]
Hypopharyngeal contractile integral	HPCI, mmHg.s. cm	HPCI quantifies contractile vigor in the hypopharyngeal region as the mean pressure multiplied by its duration and length.	Abnormal criteria <22.0 [1]<br>47.82 [41.96–53.67] [3]
Hypopharyngeal peak pressure	HPeakP, mmHg	HPeakP quantifies contractile vigor in the hypopharyngeal space–time box as the mean of pressure peaks along its length.	Abnormal criteria <61.6 [1]

Data summarized from previous reports (Supplementary References 1–3).

Abbreviations: UES, upper esophageal sphincter.

Supplementary References

1. Omari T, Ross A, Schar M, et al. Effect of thickened fluids on swallowing function in oropharyngeal dysphagia: impact of shear rheology and disorder subtype. Neurogastroenterol Motil 2025;37:e15003.
2. Walters RK, Gudipudi R, Gordis T, Davidson K, Nguyen SA, O'Rourke AK. A Systematic review of pharyngeal high-resolution manometry normative data. Am J Speech Lang Pathol 2024;33:1059-1068.
3. Jones CA, Lagus JF, Abdelhalim SM, Osborn CM, Colevas SM, McCulloch TM. Normative high-resolution pharyngeal manometry: impact of age, size of system, and sex on primary metrics and pressure stability. Dysphagia 2024;39:648-665.
